# Dietary Mixture of *Astragalus mongholicus* and *Codonopsis pilosula* Improved the Growth, Immunity, and Lipid Metabolism in the Largemouth Bass *Micropterus salmoides*

**DOI:** 10.1155/anu/2780291

**Published:** 2026-11-02

**Authors:** Qincheng Huang, Wen Hu, Kai Luo, Weihua Gao, Chenglong Wu, Bin Yin, Fuxian Zhang, Chang Ye, Yongbo Wang

**Affiliations:** ^1^ Hubei Key Laboratory of Waterlogging Disaster and Agricultural Use of Wetland Yangtze University Jingzhou 434025 China yangtzeu.edu.cn; ^2^ College of Life Science Huzhou Normal University Huzhou China; ^3^ Zhuhai Modern Agriculture Development Center Zhuhai 519000 China

**Keywords:** antioxidant ability, *Astragalus mongholicus*, *Codonopsis pilosula*, immunity, lipid metabolism, *Micropterus salmoides*

## Abstract

This study investigated the effects of dietary supplementation with a mixture of *Astragalus mongholicus* and *Codonopsis pilosula* extracts (QSY) on growth, immunity, antioxidant capacity, and lipid metabolism in juvenile largemouth bass (*Micropterus salmoides*). Fish (initial weight, 10.33 ± 0.07 g) were fed diets containing graded QSY levels (0%, 0.05%, 0.1%, and 0.2%) for 72 days. The results demonstrated that dietary supplementation with 0.1% QSY significantly improved growth performance, feed utilization, and nutrient apparent digestibility. Compared with the control diet, dietary 0.1% QSY enhanced intestinal function by increasing villus length (VL), villus width (VW), wall thickness (WT), and trypsin activity. Dietary 0.1% QSY could enhance immune‐related enzyme activity and upregulate the expression of anti‐inflammatory cytokines, thereby improving the survival rate of largemouth bass following a challenge with *Aeromonas veronii*. Meanwhile, dietary 0.1% QSY regulated the nuclear factor erythroid 2‐related factor 2/kelch‐like ECH‐associated protein 1 (Nrf2‐Keap1) signaling axis at the transcriptional level, hence enhancing antioxidant enzyme activities in the liver, intestine, and serum, while reducing liver damage and attenuating lipid peroxidation levels in various tissues. Furthermore, compared with the control group, dietary 0.1% QSY reduced hepatic lipid deposition through regulating lipid metabolism, specifically by promoting fatty acid β‐oxidation while inhibiting lipogenesis. However, 0.2% QSY exhibited adverse effects on the growth and survival rate against *Aeromonas veronii* challenge, suggesting excessive addition should be avoided. In conclusion, appropriate QSY supplementation (0.1%) effectively enhanced growth, immunity, and disease resistance in *M*. *salmoides*, supporting its potential as a functional feed additive in aquaculture.

## 1. Introduction

Largemouth bass (*Micropterus salmoides*, *M*. *salmoides*), native to the Mississippi River basin, United States, has become a crucial aquaculture and fishing species in the world [1]. *M*. *salmoides* has expanded rapidly due to easily achievable farming conditions, fast growth rate, and desirable meat quality, and its yield reached more than 900,000 tons in China [1, 2]. Intensive production environments often exert an immunosuppressive effect on *M*. *salmoides*, largely mediated by overcrowding and deteriorating water quality parameters, thereby fostering outbreaks of bacterial, viral, and parasitic diseases [3, 4]. Antibiotics are extensively administered in aquaculture to bolster disease resistance in aquatic animals [5]. However, their excessive use has led to the emergence of antimicrobial resistance, posing risks to animal welfare, environmental integrity, and public health [6]. Hence, there is an urgent need to establish environmentally sustainable approaches to counteract bacterial diseases by boosting the immunity of *M. salmoides*.

As natural compounds, plant extracts are obtained from various botanical sources, including roots, stems, leaves, flowers, seeds, and bark [7]. *Astragalus* species (primarily *A. membranaceus* and *A. mongholicus*) and *Codonopsis pilosula* (*C. pilosula*) are Chinese medicinal herbs, which share common features in promoting the growth and immunity of aquatic animals [8–10]. The extract of *Astragalus* polysaccharide possesses multifaceted bioactivities, encompassing antimicrobial, antioxidant, and hypolipidemic functions [9]. Similarly, *C. pilosula* polysaccharides have been reported to possess immunostimulatory effects and show potential in alleviating intestinal inflammation [8, 11]. It has been reported that dietary extract of *Astragalus* or *C. pilosula* improved the growth and immunity of red swamp crayfish (*Procambarus clarkii*) [8], white shrimp (*Litopenaeus vannamei*) [9], and yellow croaker (*Larimichthys crocea*) [12]. For functional enhancement, compound formulation of Chinese herbs has been demonstrated to be a more effective strategy. For example, a blend of 0.1% *Astragalus* polysaccharides and 0.05% *Forsythia suspensa* extract outperforms *Astragalus* polysaccharides alone in enhancing growth, digestion, and antioxidant status in large yellow croaker [12]. *Astragalus*, *Lycium barbarum*, *Rhizoma polygonate*, and *C. pilosula* in proportions (7:8:3:2) improved the growth, immunity, and lipid metabolism of Chinese soft‐shelled turtles *Pelodiscus sinensis* [13]. Supplementation of *Astragalus* and *C. pilosula* extract mixture promoted growth and immunity in pigs [14]. At present, little research has been conducted on the application of the combined product of *C. pilosula* and *Astragalus* extract mixture in aquatic animals. Whether the results from mammalian studies are applicable to the economically important largemouth bass is currently unknown.

In addition, the maintenance of lipid metabolic homeostasis is closely associated with the normal immune status of aquatic animals [15, 16]. Extracts of *Astragalus and C. pilosula* have been reported to improve lipid metabolism in animals [17, 18]. Currently, no studies have been reported on the effects of *Astragalus* extract or *C. pilosula* extract on lipid metabolism in *M*. *salmoides*. Hence, this study is designed to investigate the effects of graded dietary levels of herbal compound medicine containing an equal proportion of *Astragalus* and *C. pilosula* extracts on the growth performance, immunity, antioxidant capacity, and lipid metabolism in *M*. *salmoides*. The findings are expected to provide basic data for the development and application of effective and green feed additives in aquaculture.

## 2. Materials and Methods

This investigation received ethical approval from the Committee on Animal Experiment Ethics at Yangtze University (Hubei, China). The handling of largemouth bass followed the standards set forth in the Chinese Guidelines for Laboratory Animal Care and Use (20201001).

### 2.1. Experimental Diets

Four experimental diets were formulated to be isonitrogenous and isolipidic using fish meal, rapeseed meal, soybean meal, and chicken meal as the primary protein sources and fish oil and soybean oil as the main lipid sources (Table 1). The roots of *Astragalus membranaceus* (Fisch.) Bge. var. *mongholicus* (Bge.) Hsiao (denoted as *A. mongholicus* throughout the text) [19] and *C. pilosula* (Franch.) Nannf. were mixed in a 1:1 ratio (*w/w*) and subjected to a whole‐extraction process involving water extraction, concentration, ethanol precipitation, and drying, ultimately yielding a compound extract powder. The *Astragalus*‐*Codonopsis* powder, designated as “Qi Shen Yuan (QSY),” was supplied by Sichuan Bencao Xingnong Biological Technology Co., Ltd. and was utilized in the present study to investigate the combined additive effects of *A. mongholicus* and *C. pilosula* on *M*. *salmoides*. The detected values of the active components in QSY are provided in Table S1 (detected by the Pony Testing International Group, Beijing). QSY was included at four graded levels in feeds: 0.00% (control group), 0.05%, 0.1%, and 0.2%. All raw ingredients were ground with a pulverizer and passed through an 80‐mesh sieve for subsequent use. According to the formulation, each ingredient was precisely weighed and mixed thoroughly by step‐wise premixing. Thereafter, oils and water were added and blended homogeneously. The uniformly mixed mash was processed into extruded pellets with a diameter of 2 mm using a twin‐screw extruder (Model TSE65, Beijing Xiandai Yanggong Mechanical Technology Development Co., Ltd.). After natural cooling, the pellets were sealed in labeled bags and stored at −20°C until use.

**Table 1:** Formulation and nutritional composition of the experimental feed (%, dry matter basis).

Ingredients (%)	0	0.05	0.1	0.2
Fish meal	40	40	40	40
Rapeseed meal	8.05	8.05	8.05	8.05
Soybean meal	20	20	20	20
Chicken powder	8	8	8	8
Wheat flour	16	16	16	16
Microcrystalline cellulose	0.55	0.5	0.45	0.35
Fish oil	1	1	1	1
Soybean oil	1	1	1	1
Soybean lecithin	2	2	2	2
Complex premix^a^	2	2	2	2
Choline chloride	0.3	0.3	0.3	0.3
Monocalcium phosphate	1	1	1	1
Yttrium oxide (Y_2_O_3_)	0.1	0.1	0.1	0.1
QSY^b^	0	0.05	0.1	0.2
Total	100	100	100	100
Proximate compositions (dry matter basis, %)				
Crude protein	50.91	50.69	49.91	50.7
Crude lipid	11.14	11.91	11.4	11
Ash	11.74	11.42	11.99	11.78

^a^Provided by Guangdong Xintun Biotechnology Co., Ltd. Vitamin A ≥ 250,000 IU; vitamin D_3_ ≥ 75,000–150,000 IU; riboflavin (B_2_) ≥ 0.75 g; vitamin K_3_ ≥ 0.25 g; pyridoxine (B_6_) ≥ 0.75 g; vitamin C ≥ 5 g; niacinamide ≥ 2 g; vitamin E ≥ 2.3 g; cyanocobalamin (B_12_) ≥ 3 mg; thiamin (B_1_) ≥ 0.48 g; choline chloride ≤ 200 g; D‐calcium pantothenate ≥ 2 g; folic acid ≥ 0.1 g; inositol ≥ 4.75 g; D‐biotin ≥ 5 mg; MgSO_4_ ≥ 6.5 g; ZnSO_4_, 6–7.5 g; FeSO_4_, 2–36 g; MnSO_4_, 0.5–5 g; CuSO_4_, 0.25–1.25 g; CoSO_4_, 35–100 mg; Ca(IO_3_)_2_, 125–300 mg; and Na_2_SeO_3_, 9–25 mg.

^b^Mixture of of *A. mongholicus* and *C. pilosula* extracts (1:1). Provided by Sichuan Bencao Xingnong Biological Technology CO., Ltd. The composition of bioactive constituents is detailed in Table S1.

### 2.2. Animals Feeding and Management

A total of 480 juvenile *M*. *salmoides* with an average initial body weight of 10.33 ± 0.07 g were obtained from Wanglong Agricultural Technology CO., Ltd., Jingzhou, Hubei. Before the experiment, the fish were acclimatized for 7 days in an indoor recirculating aquaculture system at the College of Animal Science and Technology, Yangtze University. During acclimation, the fish were fed a control diet to adapt to both the rearing environment and the experimental feeding regime. Feeding was suspended 24 h before the start of the formal trial.

At the beginning of the experiment, fish exhibiting an intact body surface, normal swimming behavior, and no apparent signs of disease were selected and randomly distributed into four treatment groups in a completely randomized design. Each group consisted of three replicates, totaling 12 cylindrical rearing tanks (radius × height: 49 cm × 78 cm) equipped with independent recirculating systems. Each tank was stocked with 40 fish. The feeding trial lasted for 72 days. Throughout the experimental period, the fish were fed to apparent satiation twice daily (08:00 and 18:00), and the amount of feed offered was recorded. The uneaten feed was collected 1 h after each feeding session. Water quality parameters were maintained as follows: temperature 27 ± 1°C, dissolved oxygen >5 mg/L, pH 7.5 ± 0.4, ammonia nitrogen <0.3 mg/L, and nitrite <0.1 mg/L.

Fecal collection was initiated 2 weeks after the start of the feeding trial and continued for 7 days, with samples being collected twice daily. After collection, the samples were rinsed to remove impurities, dried to a constant weight at 60°C, ground, sieved, and stored at −20°C for subsequent determination of Y_2_O_3_ content (detected by Supervision and Testing Center for Nonferrous Metals Industry CO., Ltd.), which was used to calculate the apparent digestibility coefficients (ADCs) of dietary nutrients.

### 2.3. Sampling

At the end of the 72‐day feeding trial, fish were fasted for 24 h and then anesthetized with MS‐222 (Sigma, USA). The number and total body weight of fish in each tank were recorded for the calculation of the survival rate and growth performance. Three fish per tank were individually weighed and measured for length to determine the condition factor (CF).

Three fish per tank were randomly selected for proximate whole‐body composition analysis. Three fish per tank were randomly used for blood collection from the caudal vein. These individuals were then immediately dissected. The entire viscera and the liver were excised and weighed separately for calculation of the viscerosomatic index (VSI) and hepatopancreas index (HSI). Subsequently, samples of liver and intestinal tissue were collected and fixed in 4% paraformaldehyde for histological examination.

The collected blood samples were allowed to clot at 4°C for 24 h, followed by centrifugation at 3000 × *g* for 10 min. The supernatant serum was separated and stored at −80°C for subsequent analysis of biochemical parameters and enzyme activities. Additionally, tissue samples (stomach, liver, and intestine) were rapidly dissected from three further fish per tank, snap‐frozen in liquid nitrogen, and stored at −80°C for later determination of enzymatic activities and gene expression analysis.

### 2.4. Challenge Assay

Upon completion of the feeding trial, 10 *M*. *salmoides* exhibiting no body surface damage and normal vitality were selected from each rearing tank for the bacterial challenge. The *Aeromonas veronii* strain used for the challenge test was provided by the Laboratory of Pathogen Prevention and Control, College of Animal Science, Yangtze University. The strain was originally isolated from diseased largemouth bass. It was stored at −80°C in glycerol and revived before use, followed by three successive passages. The bacteria were cultured in brain heart infusion (BHI) medium at 28°C with shaking at 180 r/min until the logarithmic growth phase. Subsequently, the cells were harvested, resuspended in sterile phosphate‐buffered saline (PBS), and adjusted to the target concentration for intraperitoneal injection challenge. Prior to the challenge, all fish were anesthetized with eugenol and then intraperitoneally injected with a suspension of *Aeromonas veronii* (LD50 = 1.48 × 10^7^ CFU/mL; injection volume = 100 µL). Following the injection, fish were returned to normal rearing conditions. During this phase, fish were provided with their respective group‐specific feeds. Mortality was recorded daily for 7 consecutive days post‐challenge, and survival rates were calculated. The survival curve and between‐group significance were generated and analyzed using GraphPad Prism 10.1.2, employing the Mantel‐Cox test.

### 2.5. Nutritional Composition Analysis

The nutritional composition of both experimental feeds and fecal samples was assessed following the AOAC [20] standard protocols. Moisture was determined by drying the samples at 105°C until a stable weight was obtained (method 934.01). The Kjeldahl method was employed to measure the crude protein content (method 988.05). Ash content was analyzed by combustion in a muffle furnace at 550°C to constant mass (method 924.05). Crude lipid content was determined by Soxhlet extraction with petroleum ether as the solvent (method 920.39). Total lipid content in whole fish and liver tissues was analyzed using the same method described above. Prior to analysis, liver samples were freeze‐dried and pulverized.

### 2.6. Physiological and Biochemical Indicators

Commercial kits supplied by Nanjing Jiancheng Bioengineering Institute were used for serum biochemical parameters, tissue antioxidant ability, and digestive enzyme activities detection. Hepatic, intestinal, and gastric samples were homogenized in ice‐cold physiological saline at a 1:9 (*w/v*) ratio and centrifuged at 12,000 × *g* for 10 min at 4°C. The resulting supernatant was collected for subsequent assays.

Hepatic, intestinal, and serum antioxidant enzyme activities detection. Catalase (CAT; Cat. No. A007‐1‐1) activity was measured with the ammonium molybdate method. Superoxide dismutase (SOD; Cat. No. A001‐3‐2) activity was determined using the WST‐1 assay. Total antioxidant capacity (T‐AOC; Cat. No. A015‐2‐1) was assessed by the ABTS method. Malondialdehyde (MDA; Cat. No. A003‐1‐2) content was quantified with the thiobarbituric acid reaction.

Intestinal and gastric digestive enzyme activities detection. Trypsin (Cat. No. A080‐2‐2), lipase (Cat. No. A054‐2‐1), and amylase (Cat. No. C016‐1‐2) activities were measured using ultraviolet colorimetry, methylresorufin‐based substrate method, and starch‐iodine colorimetry, respectively.

Serum albumin (ALB; Cat. No. A028‐2‐1), lysozyme (LZM; Cat. No. A050‐1‐1), alkaline phosphatase (AKP; Cat. No. A059‐2‐2), acid phosphatase (ACP; Cat. No. A060‐2‐2), alanine aminotransferase (ALT; Cat. No. C009‐2‐1) and aspartate aminotransferase (AST; Cat. No. C010‐2‐1) levels were determined with a microplate enzymatic method. Total cholesterol (T‐CHO; Cat. No. A111‐1‐1) and triglyceride (TG; Cat. No. A110‐1‐1) contents were analyzed using the GPO‐PAP method. High‐density lipoprotein cholesterol (HDLC; Cat. No. A112‐1‐1) and low‐density lipoprotein cholesterol (LDLC; Cat. No. A113‐1‐1) were quantified with a direct two‐reagent assay.

### 2.7. Molecular Experiment

Total RNA was extracted from hepatic and intestinal tissues of sampled fish with the Trizol reagent (Beyotime, China). RNA purity and concentration were assessed using a microspectrophotometer (Nano‐400 A, Aosheng Instruments, China). Reverse transcription was performed on the total RNA using the MonScript RTIII Super Mix with dsDNase (Two‐Step) kit (Monna Bio, China) to generate complementary DNA (cDNA). Primers targeting genes of interest were designed in Premier 5.0 and commercially synthesized by Sangon Biotech (Shanghai, China; Table 2). Quantitative real‐time PCR (RT‐qPCR) amplification was carried out on a StepOnePlus RT‐qPCR System (Applied Biosystems, USA) in 20 μL reactions consisting of 10 μL MonAmp ChemoHS qPCR Mix (YiSheng Biological Technology CO., Ltd., China), 0.4 μL forward primer, 0.4 μL reverse primer, 0.2 μL High ROX Dye (100×), 2 μL cDNA template, and 7 μL nuclease‐free water. The PCR conditions comprised an initial denaturation at 95°C for 10 min, followed by 40 cycles of 95°C for 10 s, 60°C for 10 s, and 72°C for 30 s. β‐actin was identified as the stable internal control. The relative expression of target genes was calculated using the 2^−ΔΔCt^ method.

**Table 2:** Primers for real‐time quantitative polymerase chain reaction (qPCR).

Gene	Forward primer	Reverse primer	Sequence
*fasn*	GGGTTGTCATGGAAATCA	CTCAAACTTGTTGGTGGC	XM_038735140.1
*srebp1*	AGGACACCAAGCCGAATG	CCAGAGGGTTGAGGGAGA	XM_038699585.1
*pparα*	AGCAGGAGCGTTGAAGGA	AGAACCCAGGGACGGACT	XM_038705497.1
*cpt1*	TTACAGGGACAAAGGGAA	CACGAACAAAGGCACAAG	XM_038705335.1
*acc1*	GCCTATGCCCACTTACCC	TGCCACCATACCAATCTCAT	XM_038709737.1
*il-10*	CGGCACAGAAATCCCAGAGC	CAGCAGGCTCACAAAATAAACATCT	XM_038696252.1
*IL-1β*	CGTGACTGACAGCAAAAAGAGG	GATGCCCAGAGCCACAGTTC	XM_038733429.1
*tnf-α*	CTTCGTCTACAGCCAGGCATCG	TTTGGCACACCGACCTCACC	XM_038710731.1
*tgf-β1*	GCTCAAAGAGAGCGAGGATG	TCCTCTACCATTCGCAATCC	XM_038693206.1
*myd88*	CTCAACCCCAAGAACACA	CGAAGATCCTCCACAATG	XM_038728827.1
*keap1*	GTGGTGGGAAGACTTATTG	TCCAGGTGCTTAGTGAGG	XM_038728593.1
*nrf2*	TCACCAAAGACAAGCGTAA	CAGGCAGATTGATAATCATAGA	XM_038720536.1
*cat*	TGCTGTCCGCTTCTCCAC	TCCCAGTTGCCCTCCTCA	XM_038704976.1
*sod1*	AGACAATACAAATGGGTGC	CTTTGCGACATTATCTGCT	XM_038708943.1
*β-actin*	TCCTCGGTATGGAGTCTTG	GTCAGCGATTCCAGGGTA	XM_038712920.1

*Note: acc1*, acetyl‐CoA carboxylase 1; *cat*, catalase; cpt1, carnitine palmitoyltransferase 1; *fasn*, fatty acid synthase; *il-10*, interleukin 10; *IL-1β*, interleukin 1 beta; *keap1*, kelch‐like ech‐associated protein 1; *myd88*, myd88 innate immune signal transduction adaptor; *nrf2*, nuclear factor, erythroid 2‐like 2; *pparα*, peroxisome proliferator activated receptor alpha; *sod1*, superoxide dismutase 1; *srebp1*, sterol regulatory element‐binding protein 1; *tgf-β1*, transforming growth factor beta 1; *tnf-α*, tumor necrosis factor alpha.

### 2.8. Morphological Structure

Fixed intestinal and liver tissues underwent dehydration, clearing, and paraffin embedding for sectioning. Intestinal sections were stained with hematoxylin and eosin (H&E). Liver sections were subjected to Oil Red O staining. Quantitative analysis of Oil Red O staining was performed using Image J software, following the method described by Mehlem et al. [21]. Morphological examination and image acquisition were performed using a light microscope (SOPTOP EX31, Shunyu Optics Technology CO., Ltd.). Intestinal structural parameters, including diameter, villus length (VL), villus width (VW), wall thickness (WT), and intestinal diameter (ID), were quantified using Image J software.

### 2.9. Computational Formula and Statistical Analysis

Weight gain rate (WGR, %) = (*W*_*t*_ − *W*_0_)/*W*_0_ × 100; specific growth rate (SGR, %/day) = 100 × (ln*W*_*t*_ − ln*W*_0_)/*t*; feed intake rate (FIR, %) = FI/(*t* × (*W*_0_ + *W*_*t*_)/2) × 100; feed conversion ratio (FCR) = FI/(*W*_*t*_ − *W*_0_); HSI (%) = *W*_1_/*W*_*t*_ × 100; visceral somatic indices (%) = *W*_2_/*W*_*t*_ × 100; CF (g/cm^3^) = *W*_*t*_/*L*_*t*_^3^ × 100; survival rate (SR, %) = *A*_2_/*A*_1_ × 100. ADC (%) = (1 − [Y_2_O_3_ content in feed/Y_2_O_3_ content in feces] × [nutrient content in feces/nutrient content in feed]) × 100%. *A*_1_, initial number of fish; *A*_2_, final fish number; *W*_0_, initial fish weight (g); *W*_*t*_, final fish weight (g); *L*_*t*_, final fish body length; *t*, experimental period (days); FI, feed intake; *W*_1_, liver weight; *W*_2_, internal organ weight.

To compare differences across experimental groups, one‐way ANOVA followed by Tukey’s post hoc test was performed in SPSS 20.0. Prior to one‐way ANOVA, the assumptions of homogeneity of variances and normal distribution were tested. Statistical significance was defined at *p*  < 0.05. All values in this study are reported as mean ± SD.

## 3. Results

### 3.1. Growth Performance and Morphological Indicators

There were significant improvements in FBW, WG, SGR, and CF in *M*. *salmoides* fed diets supplemented with 0.05% and 0.1% QSY compared to the control group (*p* < 0.05, Table 3). The best performance in terms of FBW, WG, and SGR was observed in fish receiving the 0.1% QSY diet (*p* < 0.05). Fish in the 0.1% QSY group showed 1.04‐, 1.04‐, and 1.02‐fold higher FBW, WG, and SGR, respectively, than those in the control group. Additionally, the lowest FR and FCR were recorded in this group (*p* < 0.05). However, when compared to the 0.1% QSY group, dietary supplementation with 0.2% QSY led to a significant reduction in FBW, WG, and SGR, along with a significant increase in FR and FCR (*p* < 0.05). There was also a significant increase in CF in QSY‐supplemented groups relative to the control (*p* < 0.05). No significant effects of dietary QSY were found on SR, VSI, or HSI (*p* > 0.05).

**Table 3:** The effect of dietary QSY on the growth performance of largemouth bass.

Indices (%)	0	0.05	0.1	0.2
IBW (g)	10.33 ± 0.07	10.33 ± 0.07	10.33 ± 0.07	10.33 ± 0.07
FBW (g)	74.69 ± 0.21^a^	75.85 ± 0.12^b^	77.55 ± 0.44^c^	75.10 ± 0.07^a^
SR (%)	97.5 ± 0.00	99.17 ± 1.44	98.33 ± 1.44	99.17 ± 1.44
WG (%)	622.81 ± 6.44^a^	634.10 ± 4.30^b^	650.49 ± 8.87^c^	629.78 ± 4.65^ab^
SGR (%/day)	2.41 ± 0.01^a^	2.43 ± 0.01^b^	2.46 ± 0.01^c^	2.42 ± 0.01^ab^
FR (%/day)	0.81 ± 0.01^a^	0.78 ± 0.01^ab^	0.73 ± 0.01^b^	0.80 ± 0.01^a^
FCR	1.00 ± 0.04^a^	0.98 ± 0.05^a^	0.89 ± 0.03^b^	0.99 ± 0.01^a^
CF (g/cm^3^)	1.64 ± 0.01^a^	1.71 ± 0.03^b^	1.74 ± 0.04^b^	1.73 ± 0.02^b^
VSI (%)	6.31 ± 0.05	6.29 ± 0.19	6.53 ± 0.04	6.62 ± 0.31
HSI (%)	1.17 ± 0.10	1.13 ± 0.02	1.23 ± 0.07	1.29 ± 0.07

*Note*: Different superscript lowercase letters in the same line denote significant differences. Data are expressed as mean ± SD, with statistical significance defined at *p* < 0.05.

Abbreviations: CF, condition factor; FBW, final body weight; FCR, feed conversion ratio; FR, feed rate; HSI, hepatopancreas index; SGR, specific growth rate; SR, survival rate; VSI, viscerosomatic index; WG, weight gain.

### 3.2. Apparent Digestibility

Compared to the control group, dietary supplementation with 0.1% QSY significantly increased the ADC of dry matter in *M*. *salmoides* (*p* < 0.05, Table 4). Furthermore, all tested QSY inclusion levels (0.05%, 0.1%, and 0.2%) markedly enhanced the ADCs of dietary crude protein and crude fat (*p* < 0.05). However, when compared to the 0.1% QSY treatment, the higher supplementation level of 0.2% QSY resulted in a significant reduction in the ADCs of both dry matter and crude fat (*p* < 0.05).

**Table 4:** The effect of QSY on the apparent digestibility of largemouth bass.

Indices	Groups			
	0	0.05	0.1	0.2
Dry matter (%)	60.39 ± 1.14^a^	61.55 ± 1.07^a^	68.35 ± 1.44^b^	63.38 ± 0.98^a^
Crude protein (%)	81.55 ± 0.53^a^	85.46 ± 0.40^b^	86.07 ± 0.64^bc^	86.90 ± 0.35^c^
Crude lipid (%)	78.19 ± 0.63^a^	81.07 ± 0.52^b^	86.47 ± 0.62^c^	82.54 ± 0.47^b^

*Note*: Data are expressed as mean ± SD with statistical significance defined at *p* < 0.05. Different superscript lowercase letters in the same line denote significant differences.

### 3.3. Digestive Enzyme Activity

Compared to the control group, dietary supplementation with QSY did not affect the activities of gastric digestive enzymes, intestinal lipase, or intestinal amylase in *M*. *salmoides* (*p* > 0.05, Table 5), but it significantly enhanced intestinal trypsin activity (*p* < 0.05). Dietary supplementation with 0.1% or 0.2% QSY significantly enhanced intestinal trypsin activity (*P* < 0.05).

**Table 5:** The effect of QSY on the digestive enzyme activities in the intestine and stomach of largemouth bass.

Items	Groups			
	0	0.05	0.1	0.2
Stomach				
trypsin (U/mgprot)	18.12 ± 2.19	18.60 ± 1.65	17.75 ± 0.84	17.84 ± 1.97
lipase (U/gprot)	0.74 ± 0.13^ab^	0.67 ± 0.06^a^	0.89 ± 0.11^b^	0.75 ± 0.12^ab^
amylase (U/mgprot)	0.068 ± 0.009	0.066 ± 0.005	0.066 ± 0.005	0.067 ± 0.003
Intestine				
trypsin (U/mgprot)	32.22 ± 1.29^a^	37.85 ± 1.01^ab^	47.55 ± 6.47^c^	43.40 ± 2.94^bc^
lipase (U/gprot)	0.67 ± 0.05	0.60 ± 0.02	0.65 ± 0.06	0.61 ± 0.02
amylase (U/mgprot)	0.68 ± 0.08	0.66 ± 0.07	0.67 ± 0.07	0.65 ± 0.06

*Note*: Data are expressed as mean ± SD with statistical significance defined at *p* < 0.05. Different superscript lowercase letters in the same line denote significant differences.

### 3.4. Intestinal Morphological Structure

Intestinal tissue sections are shown in Figure 1. Intestinal histomorphology was quantified (Table 6). The results revealed that compared to the control group, dietary supplementation with 0.1% and 0.2% QSY significantly increased the VL, VW, and WT in largemouth bass (*p* < 0.05). In contrast, the ID was not affected by QSY supplementation (*p* > 0.05).

**Figure 1:**
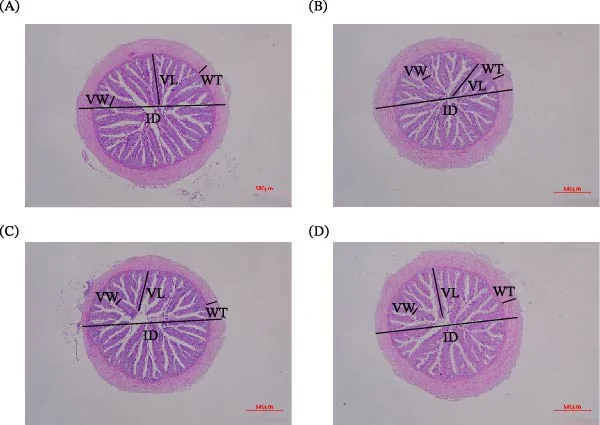
Effect of dietary QSY supplementation on the intestinal morphology of largemouth bass. ID, intestinal diameter; VL, villus length; VW, villus width; WT, wall thickness. The scale bar represents 500 µm. (A–D) represent intestinal tissue sections from largemouth bass fed the control diet, and diets supplemented with 0.05%, 0.1%, and 0.2% QSY, respectively.

**Table 6:** Effect of dietary QSY on the quantitative analysis of intestinal morphology of largemouth bass.

Indices (μm)	Groups			
	0	0.05	0.1	0.2
VL	611.03 ± 63.42^a^	649.96 ± 31.02^ab^	669.87 ± 48.90^bc^	695.07 ± 73.33^b^
VW	130.24 ± 7.33^a^	137.26 ± 2.58^ab^	145.30 ± 5.88^c^	144.27 ± 1.43^bc^
WT	171.34 ± 11.30^a^	195.83 ± 36.04^ab^	214.56 ± 32.46^b^	219.18 ± 25.27^b^
ID	1933.18 ± 24.72	1932.23 ± 101.13	1937.45 ± 24.94	1931.77 ± 66.02

*Note*: Different superscript lowercase letters in the same line denote significant differences. Data are expressed as mean ± SD with statistical significance defined at *p*  < 0.05.

Abbreviations: ID, intestinal diameter; VL, villus length; VW, villus width; WT, wall thickness.

### 3.5. Serum Biochemistry

Dietary QSY supplementation at any level significantly reduced serum ALT level in *M*. *salmoides* compared to the control (*p* < 0.05, Table 7). The 0.1% QSY group showed a marked reduction in serum AST and TG levels (*p* < 0.05). Furthermore, there was a significant increase in serum AKP activity with 0.1% and 0.2% QSY supplementation (*p* < 0.05). Largemouth bass fed the 0.1% QSY diet exhibited significantly higher serum HDLC content compared to those fed the control and 0.05% QSY diets (*p* < 0.05). No significant alterations were observed in the serum biochemical indicators, including ALB, ACP, LZM, T‐CHO, and LDLC of largemouth bass following dietary QSY supplementation, relative to the control group (*p* > 0.05).

**Table 7:** The influence of QSY on the serum biochemistry indices of largemouth bass.

Items	Groups			
	0	0.05	0.1	0.2
ALB (g/L)	16.01 ± 1.46	16.33 ± 0.89	17.29 ± 0.44	16.24 ± 0.68
ALT (U/L)	6.62 ± 0.88^b^	3.63 ± 0.70^a^	3.54 ± 0.13^a^	3.92 ± 0.93^a^
AST (U/L)	19.82 ± 1.59^b^	18.33 ± 2.26^ab^	16.06 ± 1.24^a^	17.23 ± 1.28^ab^
ACP (King unit/100 mL)	6.79 ± 2.12	7.82 ± 1.10	9.40 ± 1.72	8.23 ± 0.42
AKP (King unit/100 mL)	7.03 ± 0.55^a^	8.46 ± 1.31^ab^	9.83 ± 0.36^b^	9.85 ± 0.67^b^
LZM (U/mL)	247.11 ± 50.41	254.22 ± 51.80	256.00 ± 78.93	238.22 ± 68.58
T‐CHO (mmol/L)	6.87 ± 0.28	6.52 ± 0.31	6.52 ± 0.82	7.34 ± 0.73
TG (mmol/L)	1.63 ± 0.01^bc^	1.59 ± 0.01^ab^	1.58 ± 0.02^a^	1.67 ± 0.01^c^
HDLC (mmol/L)	2.08 ± 0.21^a^	2.20 ± 0.09^a^	2.80 ± 0.18^b^	2.41 ± 0.22^ab^
LDLC (mmol/L)	2.77 ± 0.41^ab^	2.60 ± 0.41^ab^	2.49 ± 0.39^a^	3.22 ± 0.10^b^

*Note*: Data are expressed as mean ± SD with statistical significance defined at *p* < 0.05. Different superscript lowercase letters in the same line denote significant differences.

Abbreviations: ACP, acid phosphatase; AKP, alkaline phosphatase; ALB, albumin; ALT, alanine aminotransferase; AST, aspartate aminotransferase; HDLC, high‐density lipoprotein cholesterol; LDLC, low‐density lipoprotein cholesterol; LZM, lysozyme; T‐CHO, total cholesterol; TG, triglyceride.

### 3.6. Antioxidant Enzyme Activity

In the intestine of largemouth bass, dietary supplementation with 0.05% and 0.1% QSY significantly increased T‐AOC compared to the control group (*p* < 0.05, Table 8). Furthermore, the 0.1% QSY group exhibited a marked enhancement in SOD activity (*p* < 0.05). Supplementation with 0.1% QSY also led to a significant reduction in MDA content relative to the control (*p* < 0.05). In contrast, the 0.2% QSY group showed a notably lower intestinal T‐AOC than the 0.1% QSY group (*p* < 0.05).

**Table 8:** Effect of dietary QSY on antioxidant ability in the intestine, liver, and serum of largemouth bass.

Items	Groups				
	0	0.05	0.1	0.2	
Intestine					
T‐AOC (mmol/gprot)	0.59 ± 0.03^a^	0.65 ± 0.01^b^	0.67 ± 0.01^c^	0.61 ± 0.02^ab^	
SOD (U/mgprot)	34.01 ± 3.59^a^	37.40 ± 1.73^ab^	41.68 ± 3.37^b^	39.02 ± 1.31^ab^	
CAT (U/mgprot)	5.12 ± 0.64	5.39 ± 0.71	5.84 ± 0.45	5.29 ± 0.20	
MDA (nmol/mgprot)	3.95 ± 0.56^b^	3.79 ± 0.19^ab^	3.01 ± 0.58^a^	3.86 ± 0.42^ab^	
Liver					
T‐AOC (mmol/gprot)	0.25 ± 0.04	0.24 ± 0.10	0.36 ± 0.17	0.36 ± 0.11	
SOD (U/mgprot)	93.76 ± 3.31^a^	101.18 ± 2.11^b^	100.94 ± 3.00^b^	96.17 ± 1.20^ab^	
CAT (U/mgprot)	19.39 ± 0.77^a^	20.42 ± 0.58^ab^	21.47 ± 0.67^b^	20.75 ± 0.64^ab^	
MDA (nmol/mgprot)	1.64 ± 0.28^b^	1.44 ± 0.07^ab^	0.92 ± 0.12^a^	1.51 ± 0.54^ab^	
Serum					
T‐AOC (U/mL)	0.40 ± 0.07^a^	0.52 ± 0.05^ab^	0.57 ± 0.04^b^	0.41 ± 0.07^a^	
SOD (U/mL)	18.21 ± 1.17^a^	18.83 ± 1.15^ab^	20.44 ± 1.17^b^	20.03 ± 0.91^ab^	
CAT (U/mL)	10.66 ± 0.40^ab^	9.44 ± 1.43^a^	15.13 ± 0.60^c^	11.29 ± 0.68^b^	
MDA (nmol/mL)	5.61 ± 0.72^bc^	4.03 ± 0.32^ab^	3.52 ± 1.28^a^	5.95 ± 0.21^c^	

*Note*: Data are expressed as mean ± SD with statistical significance defined at *p* < 0.05. Different superscript lowercase letters in the same line denote significant differences.

Abbreviations: GPx, glutathione peroxidase; MDA, malondialdehyde; SOD, superoxide dismutase; T‐AOC, total antioxidant capacity.

In the liver of largemouth bass, both 0.05% and 0.1% QSY increased SOD activity, while CAT activity was enhanced only in the 0.1% group relative to the control (*p* < 0.05). The hepatic MDA content was significantly lower in largemouth bass fed 0.1% QSY supplemented diet than in those fed the control diet (*p* < 0.05).

In the serum of largemouth bass, 0.1% QSY group demonstrated higher T‐AOC than both the control and 0.2% QSY groups (*p* < 0.05). This group also showed increased SOD and CAT activities alongside reduced MDA content compared to the control (*p* < 0.05). Dietary supplementation with 0.2% QSY significantly increased serum MDA content in largemouth bass compared to the 0.1% QSY group (*p* < 0.05).

### 3.7. Immune‐Related Gene Expression in the Liver

Compared with the control, dietary QSY supplementation at all supplemented concentrations significantly enhanced the hepatic mRNA levels of *il-10* and *tgf-β1* in largemouth bass (*p* < 0.05, Figure 2). No significant alterations in the expression of *myd88*, *il-1*β, or *tnf-α* were observed in fish fed either the control, 0.05%, or 0.1% QSY diets (*p* > 0.05). However, the highest supplementation level (0.2% QSY) induced a significant upregulation of hepatic *il-1β* and *tnf-α* compared to the 0.05% and 0.1% QSY groups (*p* < 0.05).

**Figure 2:**
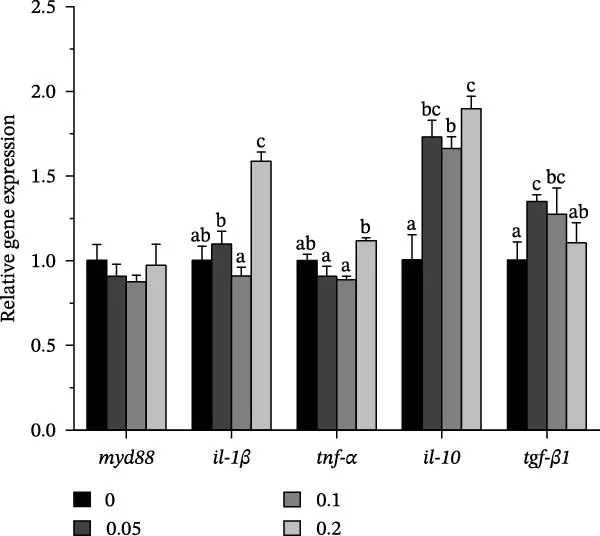
Effect of dietary QSY supplementation on the expression of immune‐related genes in the liver of largemouth bass. Bars were marked with different lowercase letters to reflect significant differences among treatments for each gene. Data are expressed as mean ± SD with statistical significance defined at *p*  < 0.05.

### 3.8. Expression of Antioxidant‐Related Genes

Dietary supplementation with 0.05% or 0.1% QSY significantly downregulated the expression of the *keap1* gene in both the liver and intestine of largemouth bass, compared to the control group (*p* < 0.05, Figure 3A,B). Concurrently, these two supplementation levels markedly upregulated the expression of *nrf2* and the *cat* and *sod1* in both examined tissues (*p* < 0.05). Notably, the 0.1% QSY supplementation yielded the most pronounced effects, producing the lowest *keap1* expression and the highest *cat* and *sod1* expression levels in the intestine, as well as the highest *nrf2* expression in the liver. However, the highest supplementation level (0.2% QSY) reversed these beneficial trends. Compared to the 0.1% QSY group, the 0.2% QSY group significantly increased intestinal *keap1* expression while suppressing the expression of *nrf2*, *cat*, and *sod1* in both the liver and intestine (*p* < 0.05).

**Figure 3:**
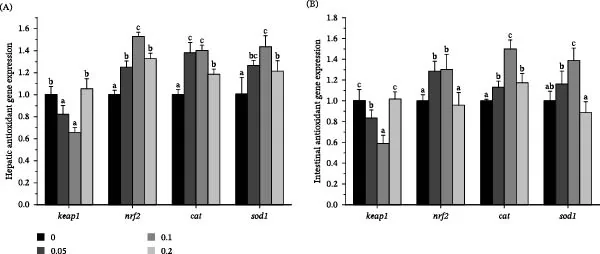
Effect of dietary QSY supplementation on the expression of antioxidative‐related genes in the liver and intestine of largemouth bass. (A) Hepatic antioxidant gene expression. (B) Intestinal antioxidant gene expression. Bars were marked with different lowercase letters to reflect significant differences (*p* < 0.05) among treatments for each gene. Data are expressed as mean ± SD.

### 3.9. Lipid Staining, Fat Deposition, and Lipid Metabolism Gene Expression

The results indicated that, compared to all other groups, largemouth bass fed the 0.1% QSY diet exhibited the highest grayscale value in hepatic oil red O staining (Figure 4A–D), indicating the lowest level of lipid deposition. Furthermore, compared to the control, whole‐body lipid content was significantly lower in fish fed the 0.1% and 0.2% QSY diets, while hepatic lipid content decreased significantly at all QSY supplementation levels (*p* < 0.05, Figure 4E,F). Specifically, hepatic TG content was markedly reduced in the 0.1% QSY group (*p* < 0.05, Figure 4G). However, the 0.2% QSY group showed a significant increase in both hepatic total lipid and TG content relative to the 0.1% QSY group (*p* < 0.05).

**Figure 4:**
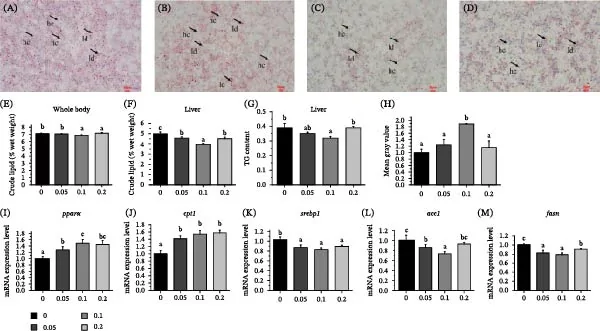
Influence of dietary QSY supplementation on fat deposition, liver oil red O staining, and hepatic lipid metabolism gene expression in largemouth bass. HC, hepatocyte; LD, lipid droplet. (A) Control, (B) 0.05% QSY, (C) 0.1% QSY, (D) 0.2% QSY; (E) whole‐body total lipid; (F) hepatic total lipid; (G) hepatic TG content; (H) quantification of the grayscale value in oil red O‐stained section, and the grayscale value is negatively correlated with the lipid content; and (I–M) Relative mRNA expression levels of hepatic lipid metabolism genes: *pparα*, *cpt1*, *srebp1*, *acc1*, and *fasn*. Data are expressed as mean ± SD. Different lowercase letters above the bars indicate significant differences (*p* < 0.05).

Regarding lipid metabolism gene expression, dietary QSY supplementation at all levels significantly upregulated the hepatic mRNA abundance of *pparα* and *cpt1* compared to the control (*p* < 0.05, Figure 4I,J). Conversely, the expression of hepatic *srebp1* (Figure 4K) and *fasn* (Figure 4M) was significantly downregulated by QSY at any concentration (*p* < 0.05). The expression of *acc1* (Figure 4L) was significantly suppressed in the 0.05% and 0.1% QSY groups (*p* < 0.05). Notably, the highest supplementation level (0.2% QSY) significantly increased the hepatic expression of *acc1* and *fasn* compared to the 0.1% QSY group (*p* < 0.05).

### 3.10. Survival Status Following Challenge Test

The results indicated that mortality in largemouth bass fed the control and 0.2% QSY diets commenced on the first day after the challenge with *Aeromonas veronii* (Figure 5). In contrast, fish receiving 0.05% and 0.1% QSY supplementation began to die on days 3 and 4 post‐challenge. Statistical analysis revealed that dietary supplementation with 0.05% or 0.1% QSY significantly enhanced the survival rate compared to the control (*p* < 0.05), although no significant difference in survival was observed between these two supplementation levels (0.05% vs. 0.1%) (*p* > 0.05). However, the 0.2% QSY group exhibited a significantly lower survival rate than the 0.1% QSY group (*p* < 0.05), and did not differ significantly from the control group (*p* > 0.05).

**Figure 5:**
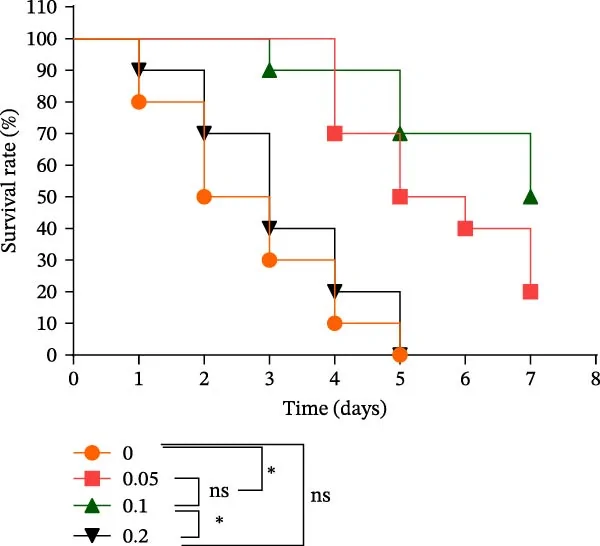
The survival rate over time of largemouth bass fed diets with varying QSY gradients after *Aeromonas veronii* challenge. ns indicates no significant difference and  ^∗^ indicates *p*  < 0.05.

## 4. Discussion

### 4.1. Dietary Mixture of *A. mongholicus* and *C. pilosula* (QSY) Promotes the Intestinal Health, Apparent Digestibility, Feed Utilization, and Growth of *M*. *salmoides*

There is limited information on the application of the *A. mongholicus* and *C*. *pilosula* mixture in aquatic animals. It has been reported that 0.1% *Astragalus* and *C. pilosula* extract mixture promoted the growth of pigs [14]. Under the current experimental conditions, supplementation levels of 0.05% and 0.1% QSY elicited a positive growth response in largemouth bass. Part of the reason can be attributed to the QSY containing an abundant content of polysaccharides. Dietary *Astragalus* polysaccharides (0.1%–0.2%) and *C*. *pilosula* polysaccharides (0.05%–0.1%) could promote the growth of Asian seabass *Lates calcarifer* and red swamp crayfish [8], respectively. The saponins present in QSY are hypothesized to be an additional growth‐promoting factor, based on previous studies showing that soya‐saponins improved the intestinal health and growth in largemouth bass [22].

Consistent with the observed growth enhancement, dietary supplementation with 0.1% QSY improved the ADC of both crude protein and crude fat. The intestine functions as the primary site for the digestion and absorption of dietary nutrients in fish, with VL, VW, and WT serving as key morphological indicators of its functional capacity [23]. The 0.1% supplementation levels significantly enhanced intestinal morphological parameters in largemouth bass, indicating improved digestive and absorptive function, which was further corroborated by a significant increase in intestinal trypsin activity. Chinese herbal medicines mixture containing *A. mongholicus* and *C. pilosula* improved the intestinal trypsin activity in *Anguilla anguilla* [24]. In contrast to the significantly improved fat ADC, dietary QSY supplementation did not alter lipase activity in the examined tissues (control vs. QSY‐added groups). This observation is not contradictory given the significant role of the gut microbiota in regulating both host growth and ADC [25–27]. *Astragalus* polysaccharides have been shown to regulate intestinal microbial homeostasis in *Litopenaeus vannamei* [28]. Future work should be made to investigate the influence of QSY on the largemouth bass gut microbiota. In addition to ameliorative ADC of nutrients, the 0.1% QSY group exhibited significantly reduced FCR and FR. It was indicated that 0.1% QSY enhanced the utilization efficiency in *M*. *salmoides*. This indicated that dietary QSY could enhance economic returns and contribute to the efficient breeding of largemouth bass. Anyhow, the present results showed that dietary mixture of *A. mongholicus* and *C. pilosula* promotes the intestinal health, apparent digestibility, feed utilization, and growth of *M*. *salmoides*.

### 4.2. Dietary Mixture of *A. mongholicus* and *C. pilosula* Improved the Hepatic Immunity and Disease Resistance of *M*. *salmoides*

Boosting the immunity of aquaculture species could mitigate the high mortality rates and economic losses associated with pathogenic infections. AST and ALT are organ‐specific biomarkers for liver, whose high activities convey the signal of liver injury [29, 30]. *Astragalus* polysaccharides have been reported to alleviate sodium chloride‐induced liver injury in mice by modulating inflammatory responses [31]. In *Lates calcarifer*, dietary supplementation with 0.1% *Astragalus* polysaccharides reduced serum AST and ALT levels [32]. Analogously, dietary QSY demonstrated a beneficial role in liver health in the present study. AKP serves as an essential component of the innate immune system in fish, exhibiting both antibacterial and anti‐inflammatory functions [33]. It has been demonstrated that dietary *Astragalus* polysaccharides (0.05%) could enhance the serum AKP activity in *Larimichthys crocea* [34]. Dietary *C. pilosula* (0.15% and 0.2%) polysaccharide increased the AKP activity in *Procambarus clarkii* [8]. Consistent with these findings, 0.1% and 0.2% levels of *Astragalus*‐*Codonopsis* mixtures also demonstrated the capacity to enhance AKP activity. To further elucidate the immunomodulatory mechanisms underlying the hepatoprotective effects of QSY, we examined the expression levels of key inflammatory cytokines. *Il-10* serves as a pivotal regulatory cytokine that orchestrates immune modulation, primarily by curbing excessive inflammation to enable effective pathogen clearance with minimal tissue damage [35]. *Tgf-β1* is a multifunctional cytokine essential for immune regulation, primarily by modulating the activity of T cells, B cells, and macrophages to control inflammatory responses and maintain immune tolerance [36]. The immunoregulatory role of *Astragalus* polysaccharides and *Codonopsis* polysaccharides has been demonstrated, respectively, in *Larimichthys crocea* and *Procambarus clarkii* [8, 37]. Administration of *C. pilosula* saponin regulated the expression of inflammatory cytokines in mice [31]. Thus, the present results suggested that 0.05% and 0.1% dietary QSY improved the hepatic immunity of largemouth bass through upregulating the expression of anti‐inflammatory factors. Intestinal inflammation can cause edema and affect the VW [38]. In the 0.1% supplementation group, pro‐inflammatory cytokines (*il-1β* and *tnf-α*) were not significantly different from the control group, whereas VW was significantly increased. Therefore, the notable changes in villus morphology in fish fed 0.1% dietary QSY were likely not inflammation‐driven, but instead reflected a beneficial and normal physiological improvement. The improved immunity was further confirmed by the high survival rate in largemouth bass fed 0.05% and 0.1% dietary QSY after *Aeromonas veronii* challenge. It has been reported that proper dietary *Astragalus* polysaccharides reduced the mortality of *Catla catla* against *Edwardsiella tarda* [39]. Therefore, it can be concluded that dietary supplementation with 0.05% and 0.1% QSY not only enhanced the hepatic immune function but also improved disease resistance in largemouth bass.

### 4.3. Dietary Mixture of *A. mongholicus* and *C. pilosula* Extracts Positively Regulated the Antioxidant Ability of *M*. *salmoides*

The enhanced immunity and growth of aquatic animals are closely related to a robust antioxidant defense system [40–42]. This system, encompassing enzymes like SOD and CAT, plays an important role in scavenging reactive oxygen species [41]. MDA is a classical marker of oxidative stress and lipid peroxidation [43]. It has been reported that dietary 0.15% *C. pilosula* polysaccharide reduced the content of hepatopancreas reactive oxygen species and increased the hemolymph SOD and T‐AOC levels in *Procambarus clarkii* [8]. Dietary 0.1% *Astragalus* polysaccharides elevated intestinal SOD activity and reduced pancreatic MDA content in *Larimichthys crocea* [44]. Saponin from *C. pilosula* could increase the serum SOD activity and reduce MDA content in mice [31]. Hence, dietary 0.1% QSY enhanced the antioxidant capacity and reduced oxidative stress of largemouth bass in the present study. Serving as the critical bridge between oxidative challenge and cellular protection, the Nrf2‐Keap1 axis translates oxidative stress signals into an adaptive transcriptional response [45]. Under basal conditions, Keap1 continuously targets Nrf2 for ubiquitin‐mediated degradation, acting as its primary negative regulator [45]. This study further found that dietary 0.1% QSY significantly upregulated the expression of *Nrf2*, *cat*, and *sod1* genes in the liver and intestine, while downregulating *keap1* expression. It was suggested that dietary 0.1% QSY might activate the Nrf2‐Keap1 signal pathway, thereby enhancing the body’s antioxidant capacity. Research in mice has proved the stimulatory effect of *Astragalus* and *C. pilosula* polysaccharides on the Nrf2‐Keap1 signal pathway [46–48]. The effect of the mixture of *A. mongholicus* and *C. pilosula* extracts on the Nrf2‐Keap1 axis was rarely reported in aquatic animals, warranting further investigation.

### 4.4. Dietary Mixture of *A. mongholicus* and *C. pilosula* Positively Regulated the Hepatic Lipid Metabolism of *M*. *salmoides*

Lipid homeostasis is closely associated with the organic oxidative stress status [31, 49]. Further investigation was made into hepatic lipid deposition of *M*. *salmoides* fed dietary QSY. It was shown that 0.1% dietary QSY reduced lipid content in the whole body and liver in the present study. Feeding 3% dietary *Astragalus* membranaceus polysaccharides markedly lowered lipid content in the whole body of *Litopenaeus vannamei* [9]. TG primarily functions in fat storage and energy supply, and elevated TG levels can increase the risk of fatty liver disease in fish [50]. Dietary 0.02% *Astragalus membranaceus* extract reduced plasma TG content in *Carassius auratus gibelio* [51]. Treatment of *Astragalus membranaceus* polysaccharide reduced plasma and liver TG content in mice fed high‐fat diets [52]. Consistent with the decrease in total liver lipids, serum and liver TG levels were also significantly reduced by the QSY‐supplemented diet. The lipid‐lowering phenotypes of QSY may be associated with its involvement in the regulation of lipid metabolism in largemouth bass. Traditional Chinese medicine has been applied for the treatment of both alcoholic and non‐alcoholic fatty liver disease due to its effectiveness in lipid metabolism regulation [53]. As a master regulator of lipid catabolism, PPARα activation represents a critical target for therapeutic strategies aimed at reducing lipid levels [54, 55]. Concurrently, CPT1 acts as the limiting enzyme in mitochondrial β‐oxidation of fatty acids and is directly transcriptionally controlled by PPARα [55]. ACC1 initiates fatty acid synthesis by producing malonyl‐CoA, which then serves as the essential substrate for FASN in the cytoplasm [56]. Critical for cellular fatty acid synthesis, the membrane‐bound transcription factor SREBP1 controls the production of multiple related enzymes, notably ACC and FASN [56]. It has been reported that 0.08% dietary *Astragalus* polysaccharides reduced and enhanced the hepatic expression of *fasn* and *ppar α*, respectively [18]. *C. pilosula* polysaccharides reduced liver fat synthesis through activating the AMP‐activated protein kinase (AMPK)/ACC/SREBP1 pathway in mice fed high‐fat diets [57]. In the present study, 0.05% and 0.1% increased transcription of *pparα* and *cpt1*, while decreased that of *srebp1*, *acc1*, and *fasn*. Therefore, dietary QSY reduced liver deposition partly through inhibiting fat synthesis and promoting fat catabolism. It was suggested that QSY may be used as a functional feed additive to optimize lipid metabolism and promote the utilization of fat in fish.

### 4.5. Excessive Addition of a Mixture of *A. mongholicus* and *C. pilosula* Does Not Yield Better Results

Evidence indicated that higher levels of dietary herbal medicine do not necessarily yield better outcomes. In *Procambarus clarkii*, compared with the optimal level (0.15%–0.2%), dietary 0.3% *C. pilosula* polysaccharide significantly weakened some immune and antioxidant indicators [8]. Relative to the optimal level of 0.1%, supplementation with dietary 0.2% or 0.4% *Astragalus membranaceus* significantly impaired the growth performance of *Cyprinus carpio* [10]. In the present study, compared with the 0.1% supplementation group, the 0.2% group showed significantly reduced growth rate and pathogen resistance in largemouth bass, suggesting that the 0.2% QSY level in the diet may be excessive. These findings highlight the need to avoid over‐adding a mixture of *A. mongholicus* and *C. pilosula* when formulating aquatic animal feeds. Based on a comprehensive evaluation of growth performance and resistance to pathogenic bacteria, the supplementation of a 0.1% mixture of *A. mongholicus* and *C. pilosula* is recommended for largemouth bass under the conditions of this experiment.

## 5. Conclusion

In conclusion, compared with the control group, dietary supplementation with 0.1% QSY (a 1:1 mixture of *Astragalus mongholicus* and *Codonopsis pilosula* extracts) significantly improved growth performance, lipid utilization, antioxidant ability, and disease resistance against *Aeromonas veronii* in juvenile largemouth bass. Mechanistically, compared with the control group, 0.1% QSY transcriptionally regulated the Nrf2‐Keap1 antioxidant pathway, upregulated anti‐inflammatory cytokines, and dually regulated lipid metabolism by promoting fatty acid β‐oxidation while inhibiting lipogenesis to reduce hepatic lipid deposition. Notably, the higher 0.2% supplementation level was detrimental, demonstrating a dose‐dependent effect of dietary QSY. Based on comprehensive evaluation, dietary 0.1% QSY is recommended as a functional phytogenic feed additive for sustainable largemouth bass aquaculture.

## Author Contributions

**Qincheng Huang:** experiment guidance, manuscript writing, funding support. **Wen Hu:** farming, detection, data analysis. **Kai Luo:** experiment guidance. **Weihua Gao:** experiment guidance, resources, funding support. **Chenglong Wu:** resources, funding support. **Chang Ye and Yongbo Wang:** sampling assistance. **Fuxian Zhang:** funding support, resources support. **Bin Yin:** resources support.

## Funding

This study was supported by the Zhejiang Provincial Natural Science Foundation of China (Grant LZ26C190001), the Hubei Key Laboratory of Waterlogging Disaster and Agricultural Use of Wetland (Grant KFG202514), the Talent Introduction Program of the Reform and Development Subsidy, Yangtze University (Grant 80210038), the Jingzhou Science and Technology Plan Project of Hubei Province of China (Grant 2025CC32), the Jingzhou Problem‐Solving Bidding Science and Technology Project of Hubei Province of China (Grant 2026JA12), and the Youth Talent Project of the Science and Technology Research Program of the Department of Education of Hubei Province (Grant Q20251301).

## Conflicts of Interest

The authors declare no conflicts of interest.

## Supporting Information

Additional supporting information can be found online in the Supporting Information section.

## Supporting information


**Supporting Information** Table S1: Actual measured content of active ingredients in QSY.

## Data Availability

The corresponding author will provide the data presented in this manuscript upon reasonable request.
