# Supplementary material for: Dietary Mixture of Astragalus mongholicus and Codonopsis pilosula Improved the Growth, Immunity, and Lipid Metabolism in the Largemouth Bass Micropterus salmoides
Source: Aquac Nutr. 2026 Nov 2;2026:2780291. doi: 10.1155/anu/2780291 (PMC13725368; doi:10.1155/anu/2780291)
Supplement: Supplementary file 1 [file ANU-2026-2780291-s001.docx]

**Supplementary Table S1 Actual measured content of active ingredients in QSY**

| Items | Content (mg/kg, wet weight) |
| --- | --- |
| Moisture content | 1.12×10^5^ |
| Total polysaccharides | 3.63×10^5^ |
| Total saponins | 6.5×10^3^ |
| Cu | 3.28 |
| Zn | 5.96 |
| Fe | 160 |
| Mn | 18.0 |
